# Supplementary material for: Conformational switching in the coiled-coil domains of a proteasomal ATPase regulates substrate processing
Source: Nat Commun. 2018 Jun 18;9:2374. doi: 10.1038/s41467-018-04731-6 (PMC6006169; doi:10.1038/s41467-018-04731-6)
Supplement: Supplementary file 1 — Supplementary Information [file 41467_2018_4731_MOESM1_ESM.pdf]

CONFORMATIONAL SWITCHING IN THE COILED-COIL DOMAINS OF A PROTEASOMAL ATPASE REGULATES SUBSTRATE PROCESSING

Snoberger et al.

Supplementary Information

Supplementary Tables 1-2, Supplementary Figures 1-8

*correspondence to
David M. Smith
dmsmith@hsc.wvu.edu

Supplementary Table 1: Summary of ATP Hydrolysis Kinetics of PAN Variants Under Oxidizing and Reducing Conditions

	Reduced				Oxidized			
	Vmax (ATP·PAN^{-1}·min^{-1})	Km [ATP] μM	Hill	CC Crosslink	Vmax (ATP·PAN^{-1}·min^{-1})	Km [ATP] μM	Hill	CC Crosslink
WT PAN	61.2 ± 2	507 ± 37	1.8 ± 0.2	None	55.9 ± 2.9	457 ± 56	1.7 ± 0.3	None
M87C	53.8 ± 1.9	413 ± 34	1.8 ± 0.2	None	52.7 ± 2.3	497 ± 48	1.8 ± 0.1	C1
59C	48.1 ± 1.4	436 ± 29	1.8 ± 0.2	None	56 ± 2	443 ± 36	1.8 ± 0.2	C1+C2
87+73C	42.2 ± 1	556 ± 29	1.8 ± 0.1	None	could not determine	>3000	could not determine	C1+C3
87+73+59C	51.3 ± 2.6	580 ± 64	1.8 ± 0.3	None	53 ± 3	637 ± 83	1.6 ± 0.3	C1+C2

Values are calculated from curves in **Supplementary Fig. 6**. Values are derived from means ± standard deviations of 3 independent experiments (n=3). 87+73C-oxidized values could not be determined because data did not fit a Michaelis-Menton Curve.

Supplementary Table 2: Normalized ATP Hydrolysis of PAN Variants

	Reduced Vmax (normalized)	Oxidized Vmax (Normalized)
WT PAN	100 ± 3.3%	100 ± 5.2%
M87C	100 ± 3.5%	107 ± 4.7%
59C	100 ± 2.9%	128 ± 4.6% **
87+73C	100 ± 2.4%	could not determine
87+73+59C	100 ± 5.1%	113 ± 6.4%

Vmax values were calculated from curves in **Supplementary Fig. 6** and values in **Supplementary Table 1**. Vmax values were normalized to WT PAN controls and divided by the reduced form of the mutant. Values are derived from means ± standard deviations of 3 independent experiments (n=3). 87+73C-oxidized values could not be determined because data did not fit a Michaelis-Menton Curve.** = $p < 0.001$

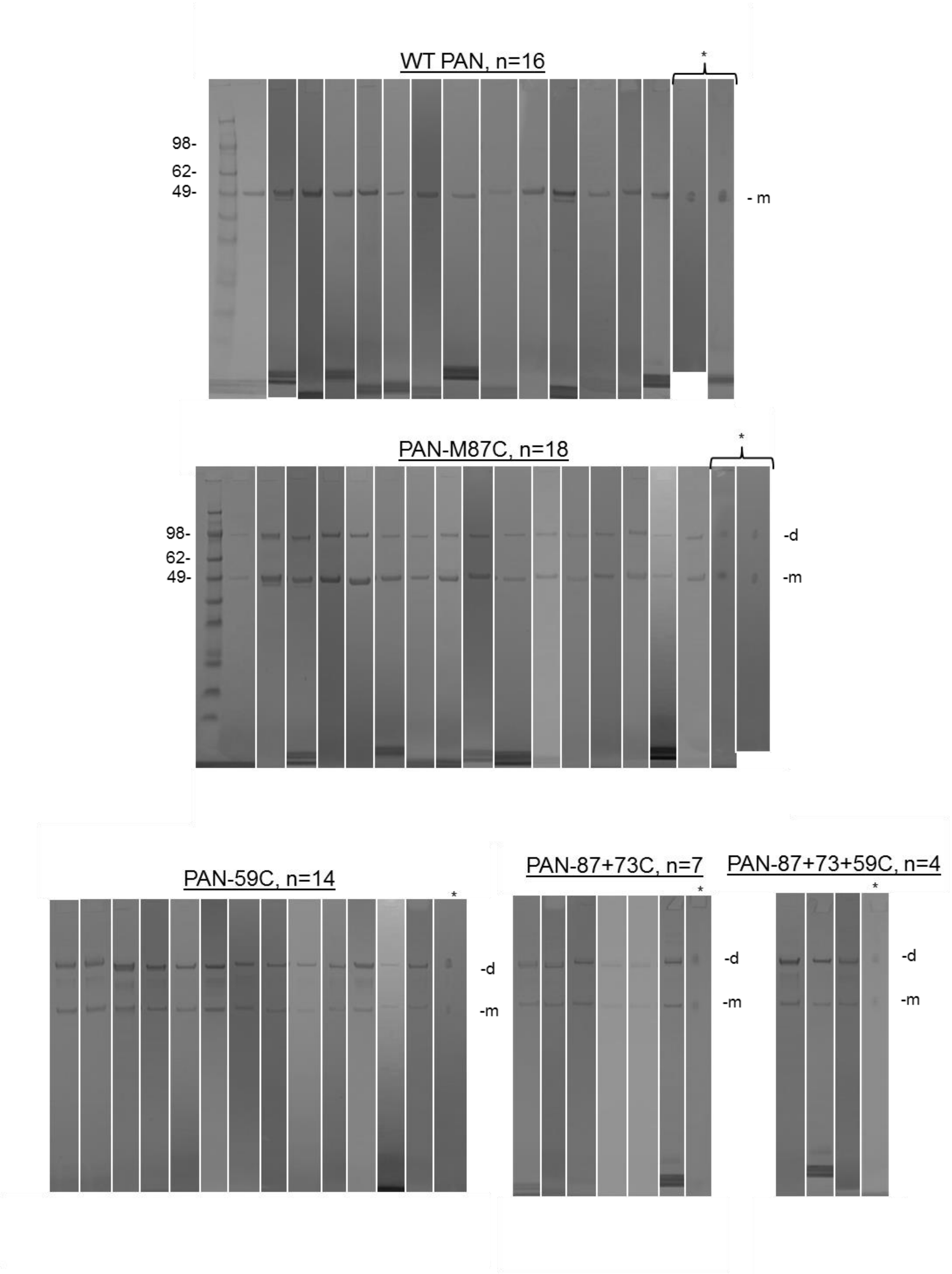

Supplementary Figure 1: Full-length lanes from nonreducing SDS-PAGE analysis for the mutants in this manuscript. ‘m’ indicates monomers and ‘d’ indicates dimers. Asterisks (*) indicate 2-dimensional gels where a Native-PAGE was run, hexameric bands excised, then analyzed via non-reducing SDS-PAGE.

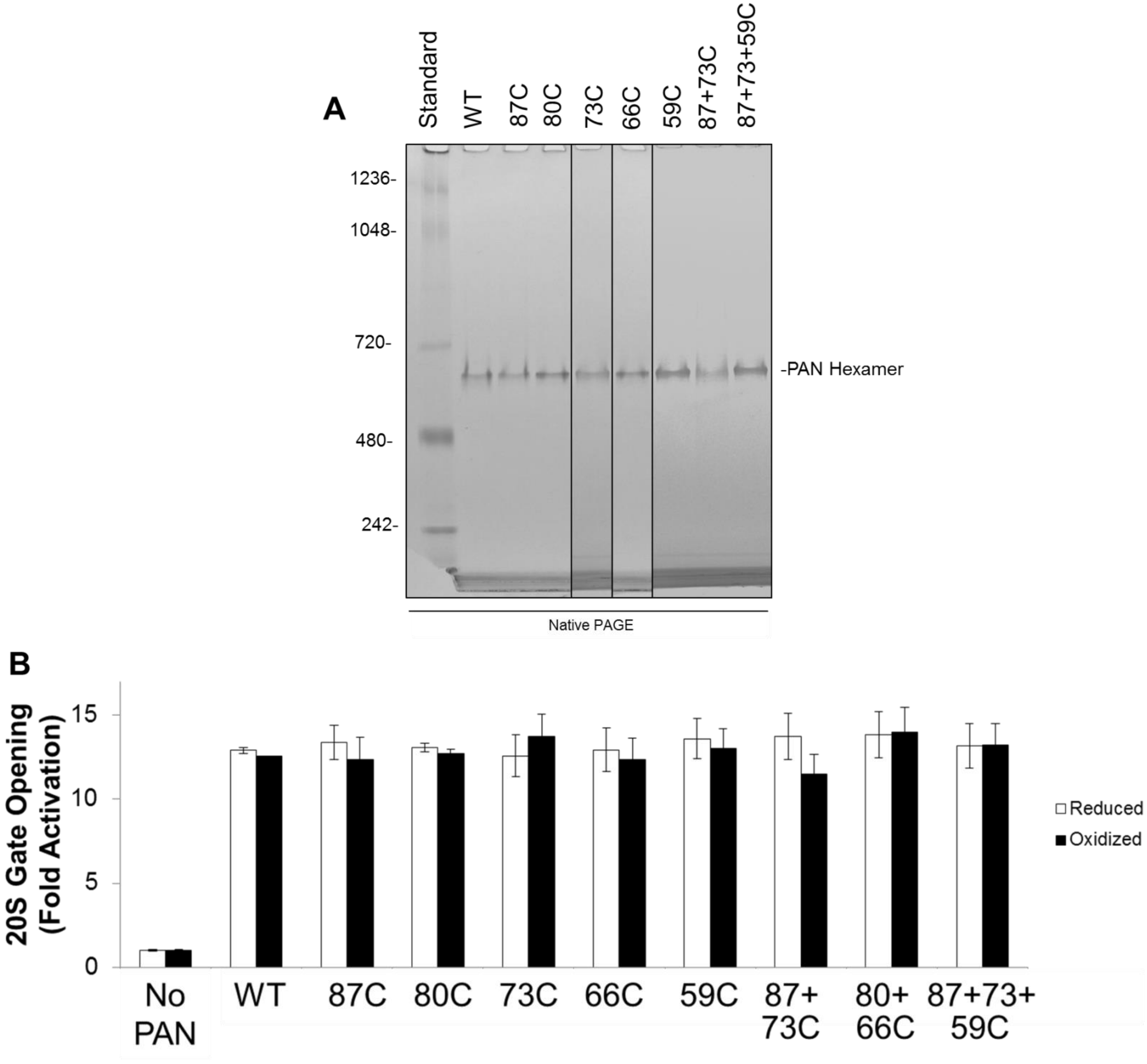

Supplementary Figure 2: PAN Mutants Retain Global Quaternary Structure and T20S Gate Opening Capacity

A) 2 µg of oxidized PAN variants were run on Native-PAGE and analyzed for hexamer formation. Experiments were performed in triplicate. Representative data is presented is from 3 non-contiguous gels. Note that PAN runs on Native gels at a higher molecular weight than expected, likely due to differences in tertiary/quaternary structure of PAN compared to the standards used. **B)** The stimulation of 20S activity (caused by PAN-induced 20S gate opening) was measured using saturating PAN and 2uM of a fluorescent reporter nonapeptide (LFP) with 10uM ATPγS and 20mM $MgCl_2$ (see methods for details). The rate of LFP hydrolysis was calculated and fold stimulation of the 20S activity by PAN is shown. 20S alone control is considered 1-fold. Bar graphs are means ± standard deviations (n=3).

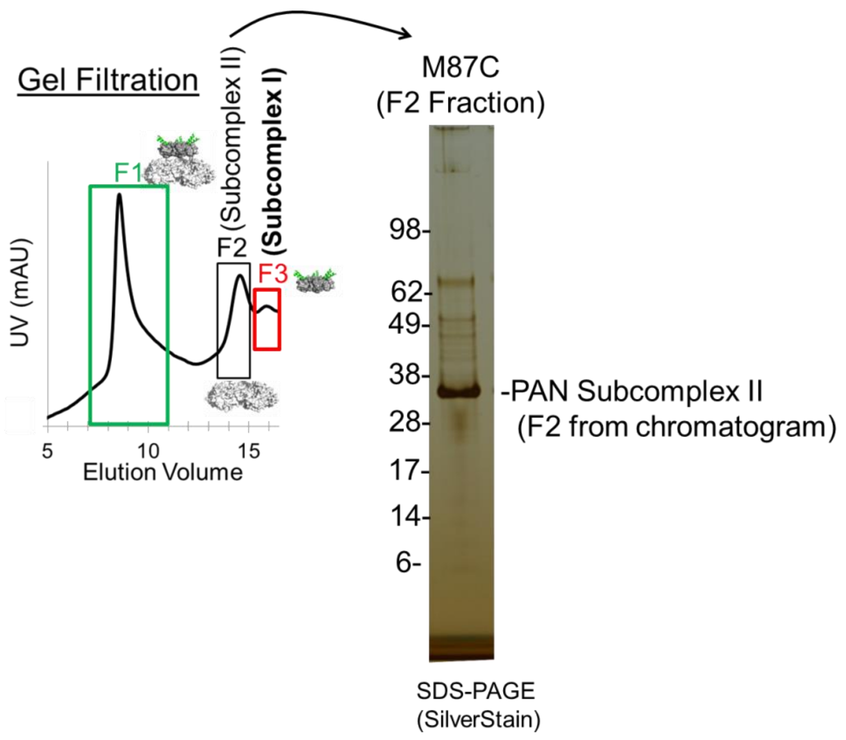

Supplementary Figure 3: SDS-PAGE of the "F2" PAN-M87C fragment. Partial proteolysis fragments of PAN were loaded onto a GE Superose 12 size exclusion column. The F2 fragment ran as a ~30-35kDa monomer, consistent with the monomeric size of PAN Subcomplex II (the AAA+ ATPase domain fragment).

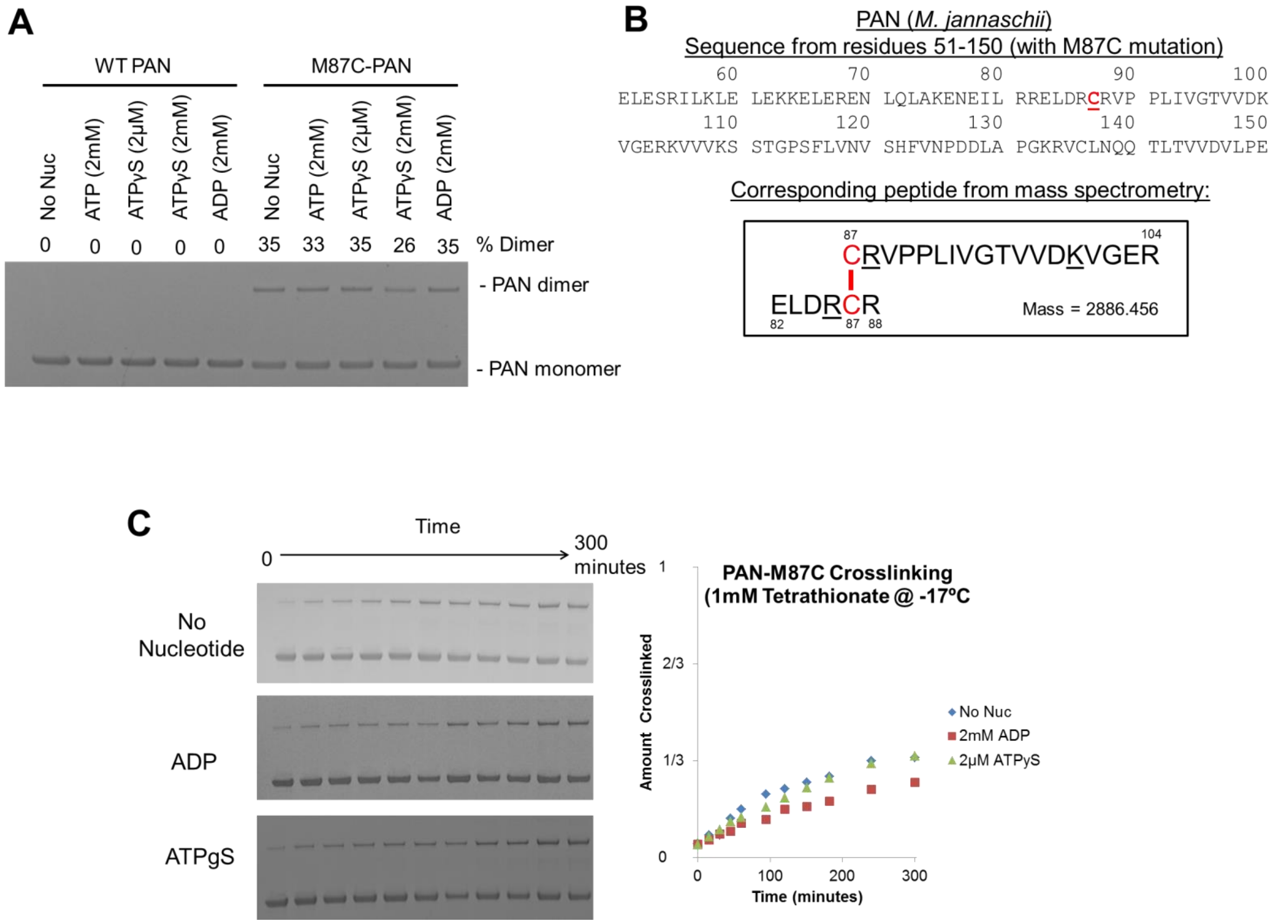

Supplementary Figure 4: Natural levels of nucleotides have little effect on disulfide crosslinking of WT PAN or PAN-M87C. A) 0.25mg/ml WT PAN or PAN-M87C was incubated for 1 hour @ room temperature with 1mM tetrathionate (and the indicated nucleotide + 10mM MgCl2). Note that the level of crosslinking is approximately the same regardless of the nucleotide bound, with the exception of high levels of ATPgS (2mM), which forces PAN into an unnatural 4-nucleotide bound conformation. **B)** Mass spectrometry of PAN-M87C reveals a peptide with a mass corresponding to a dimer with a disulfide crosslink at residue 87. **(top)** Sequence of PAN (M. jannaschii) from residues 51-150. The 87th residue mutated to cysteine is indicated in red. **(bottom)** A peptide of Mass = 2886.456 Da was found in the oxidized sample, which corresponds to 2 fragments of PAN that had been crosslinked at residue M87C. The fragment from the first monomer was from 87-104, and the peptide from second PAN monomer from 82-88. Note that both of these crosslinked fragments have missed trypsin cleavage sites (underlined), which is expected to occur when a disulfide bond occludes trypsin's access to these cut sites. **C)** PAN-M87C crosslinking timecourse. 0.25mg/ml PAN-M87C was reduced using 1mM dithiothreitol, desalted, and then incubated at (-17ºC) for 0-300 minutes with 1mM tetrathionate and the indicated nucleotides. -17º temperatures were achieved with 11% NaCl in Ice Water (w/w), and 50% glycerol was used in samples to prevent freezing of samples. Left panel is raw SDS-PAGE data of these experiments, right panel is quantification of SDS-PAGE.

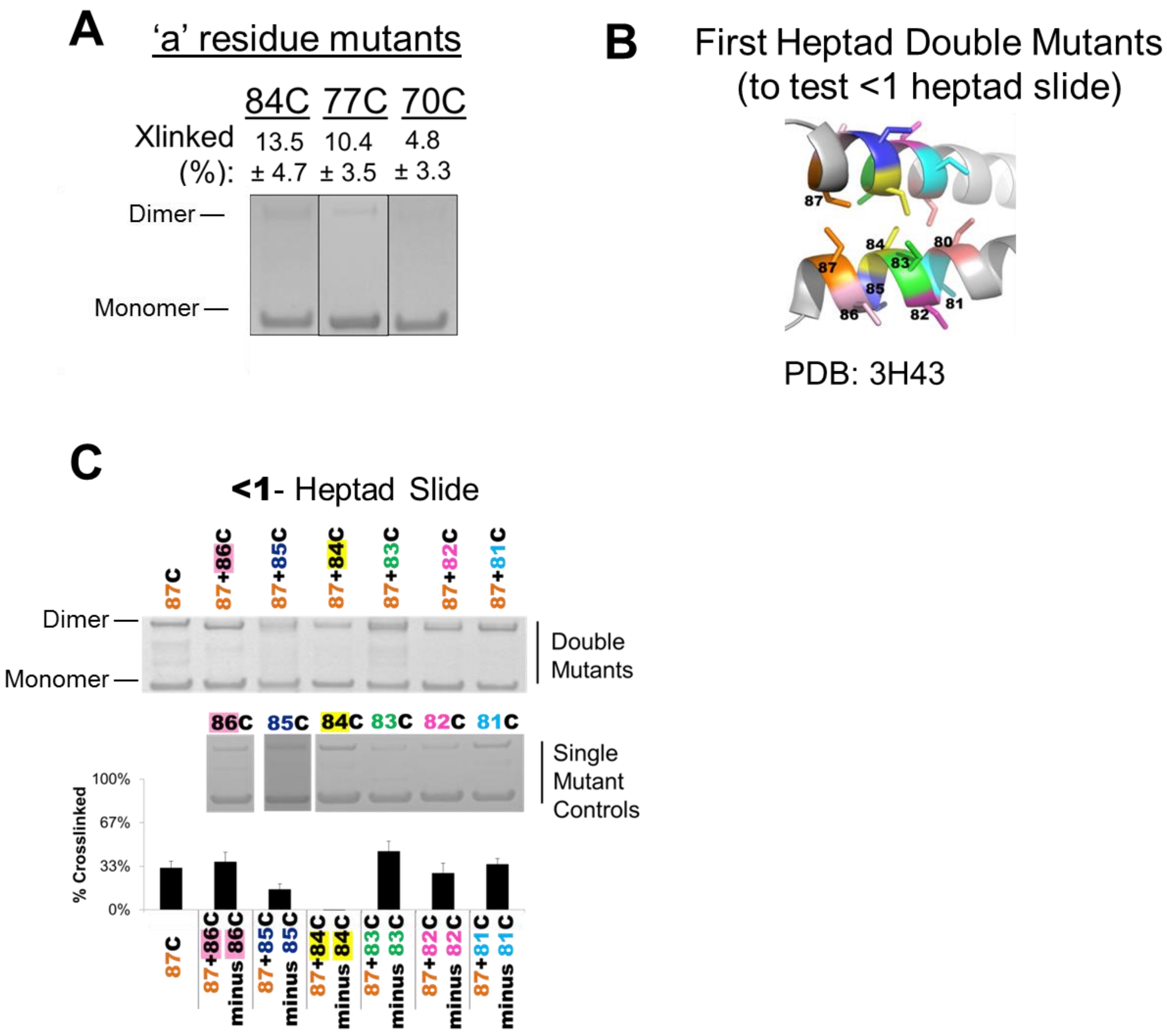

Supplementary Figure 5: PAN does not adopt a partial heptad slide.
A) Residues in the 'a' position of the heptad repeat were mutated to cysteines and subjected to crosslinking and SDS-PAGE analysis. Values presented are means ± standard deviations (n=3). **B)** Crosslinking strategy to test every possible registry shift at less than 1 heptad. Point mutations of each residue in the first heptad were generated either alone (single mutants) or with residue M87C (orange, double mutants). **C)** The indicated double mutants and their single mutant controls were subjected to oxidizing conditions (1mM tetrathionate), desalted, and run on SDS-PAGE followed by coomassie staining. These mutants can crosslink in-register and in register slides of <1 heptad. This allowed the analysis of the level of crosslinking contributed by C1 (in-register CC) plus the level of crosslinking contributed by an out-of-register CC, since single mutants can only crosslink in-register CCs. Note: some of the double mutants have less than 33% crosslinking after the background single mutant control is subtracted, likely due to destabilization of the CC. Bar graphs represent the amount of crosslinked PAN less the single mutants controls (87C- n=18, n=3 for all others)

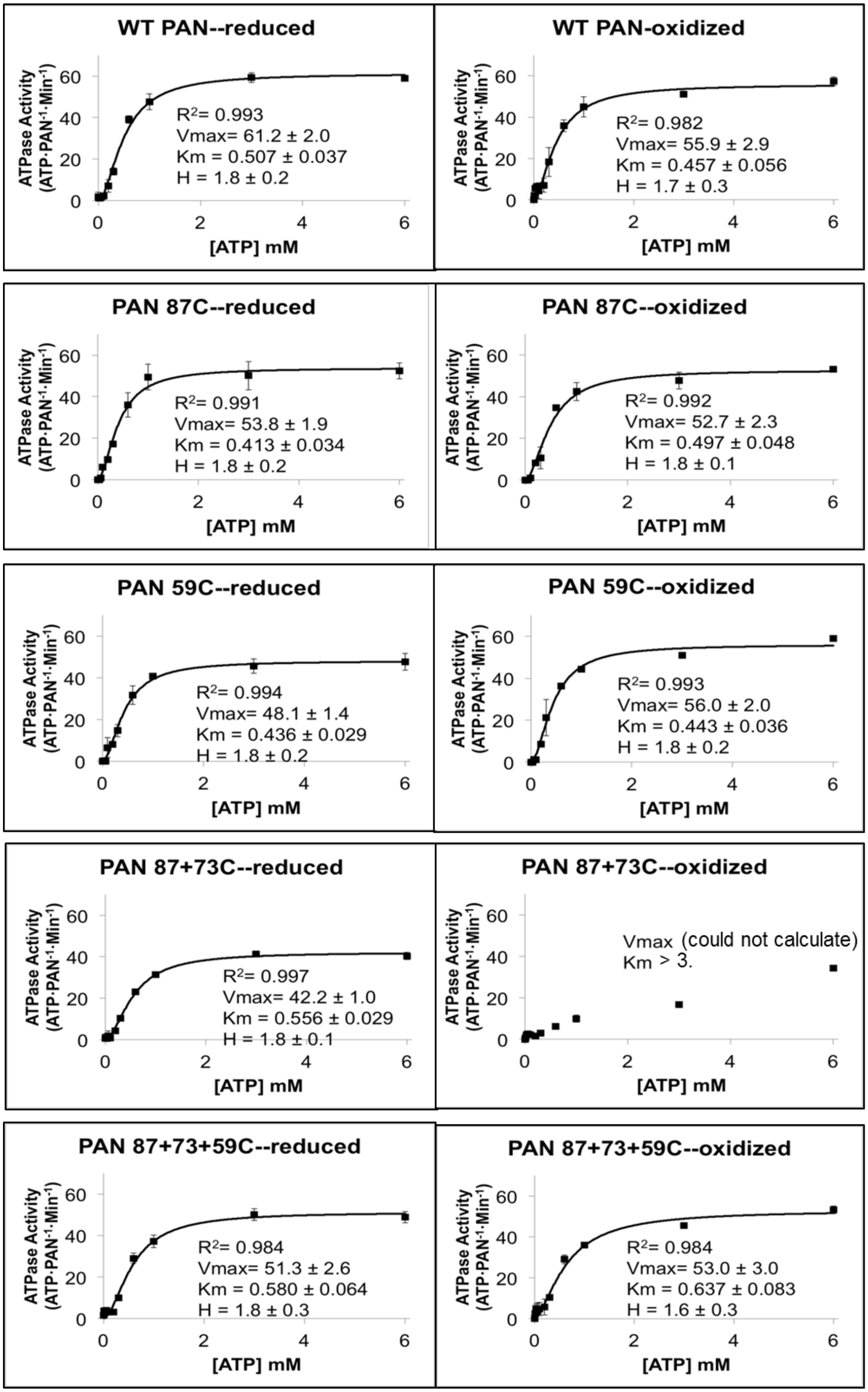

Supplementary Figure 6: ATP hydrolysis kinetics of PAN mutants under oxidizing and reducing conditions

Increasing amounts of ATP (0-6 mM) were added to PAN variant (0.05 μM) under oxidizing and reducing conditions and ATPase activity was measured via NADH-coupled assay (see methods). Data points are means ± standard deviations (n=3). Data was fit to 3 parameter Michaelis-Menten curve and Vmax, Km, and Hill coefficients were extracted and are shown. The PAN 87+73C-oxidized variant did not fit a Michaelis-Menten curve.

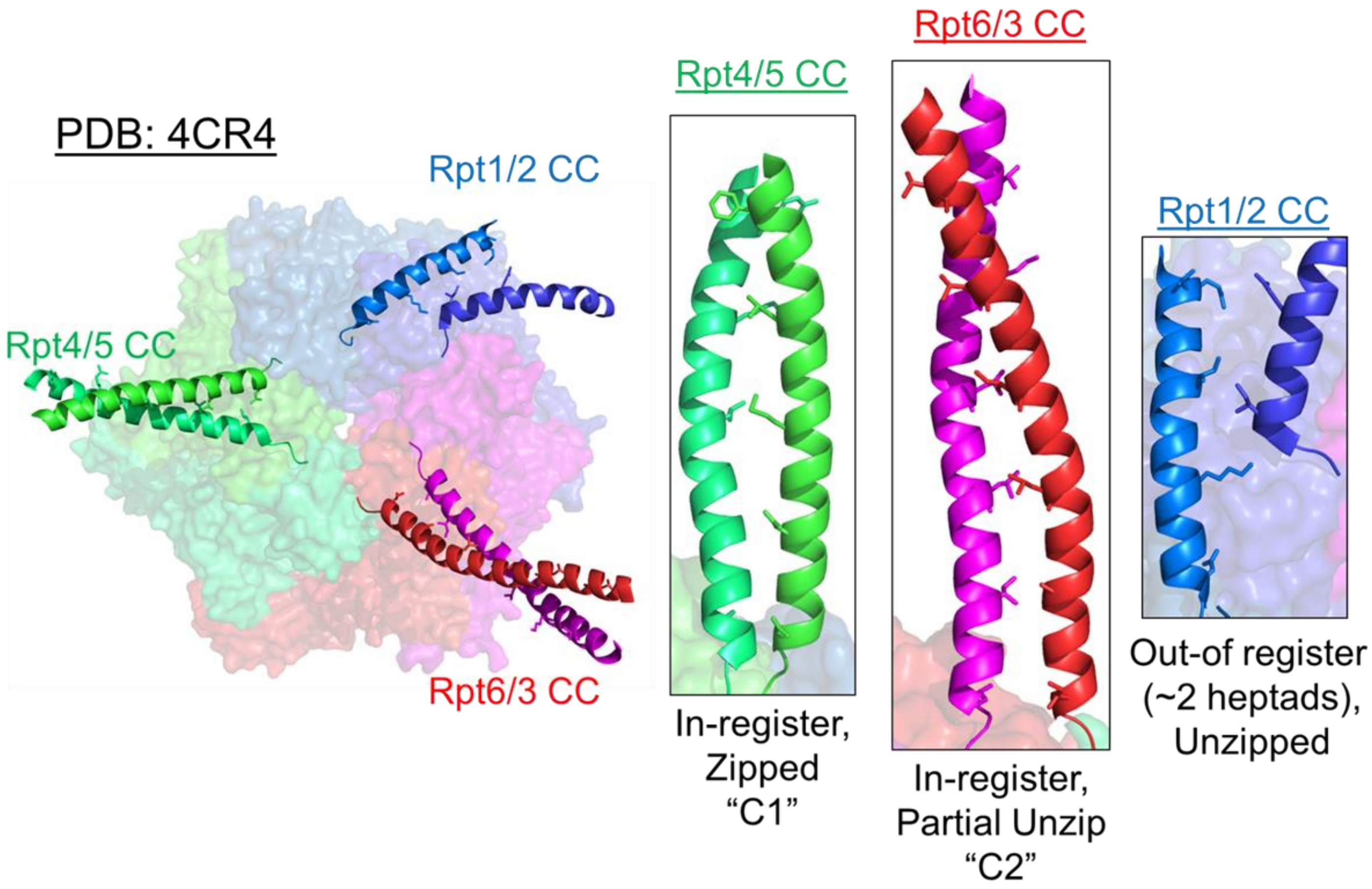

Supplementary Figure 7: Asymmetric coiled-coil conformations are also observed in the 26S ATPases (PDB: 4CR4).

In the 4CR4 cryo-EM structure, the Rpt4/5 CC is in-register and mostly zipped (C1-like), the Rpt6/3 CC is in-register and partially unzipped (C2-like), and the Rpt1/2 CC is 2 heptads out-of-register (C3-like), but appears unzipped, consistent with State #2 ("activated"), where the C1 and C2 CC conformations are crosslinkable, but not the C3 CC conformation.

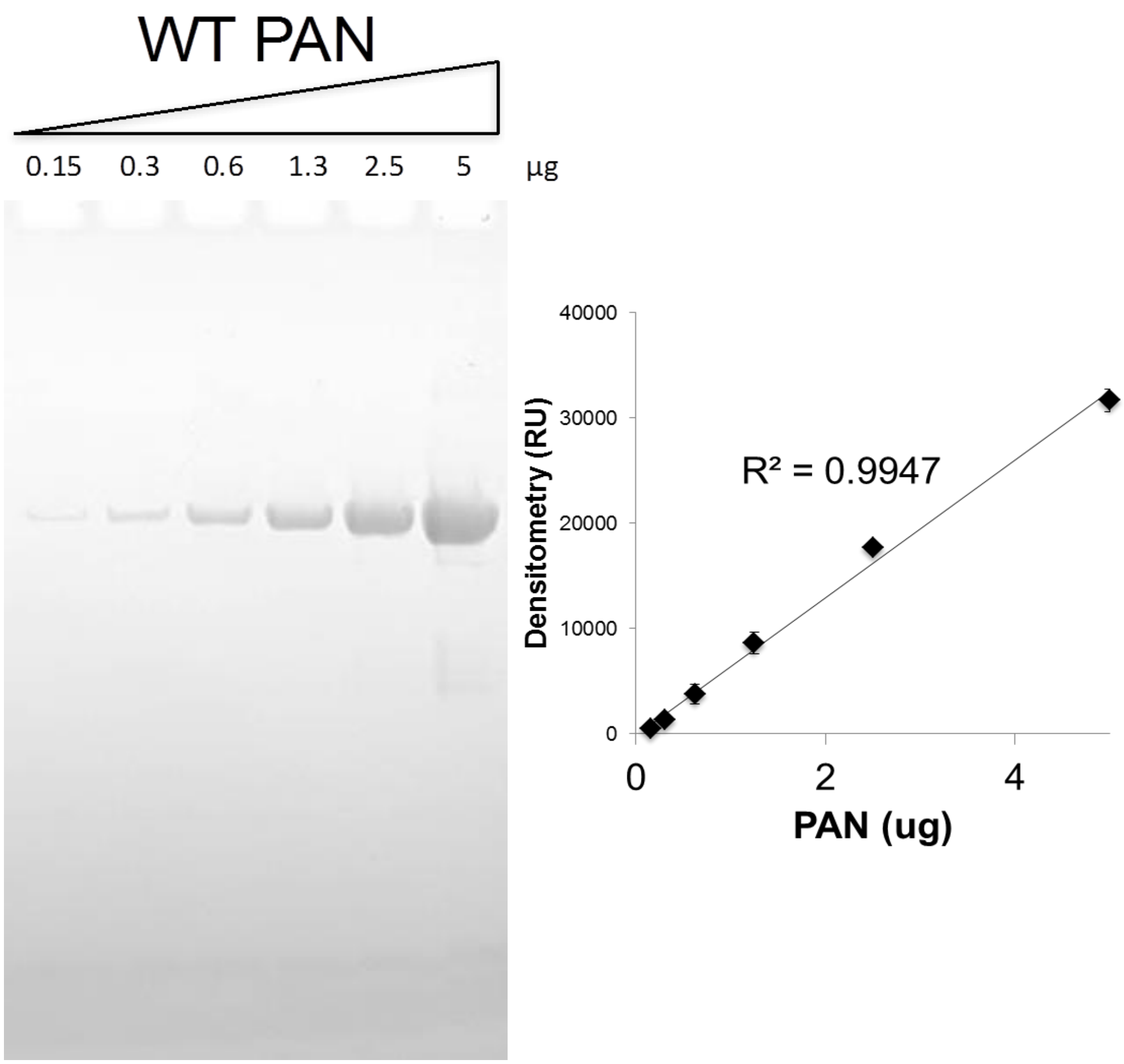

Supplementary Figure 8: WT PAN dose response on SDS-PAGE.

SDS-PAGE analysis of WT PAN dose response. Quantification (bottom) shows that densitometry increases linearly with PAN from 0.15-5 μg (R^2 = 0.99474). Values are means ± standard deviations of 3 independent experiments (n=3).
